# Longitudinal Performance Changes in Transgender Women Athletes Pre and Post Gender Affirming Hormone Therapy

**DOI:** 10.1002/ejsc.70036

**Published:** 2025-08-13

**Authors:** Joanna Harper, Richard C. Blagrove, Eugenie Hunsicker, Gemma L. Witcomb, Richard A. Ferguson, Emma O'Donnell

**Affiliations:** ^1^ School of Sport Exercise and Health Sciences Loughborough University Loughborough UK; ^2^ School of Science Loughborough University Loughborough UK

**Keywords:** hormones, runners, swimmer, testosterone, transgender

## Abstract

The aim of this study was to evaluate athletic performance and training in transgender women (TW) athletes competing in running and swimming both pre and post gender affirming hormone therapy (GAHT). Using survey methods, 9 TW runners and 1 TW swimmer provided independently verified retrospective data for race times, training volume, height, body mass, and testosterone, oestrogen and haemoglobin concentrations and pre‐GAHT and post‐GAHT. Prospective data were collected for a further 12 months. Changes in performance and age‐grade scores (AGs) for runners and FINA scores for the swimmer were calculated. For runners, pre‐GAHT and post‐GAHT differences in AGs were adjusted based on training differences over time. Post‐GAHT, testosterone concentrations in TW (1.10 ± 0.52 nmol·L^−1^) were female typical. Average race time for the runners increased by 14.6 ± 5.6% after 31 ± 23 months (range 5–86 months) of GAHT. Changes in training were positively associated with changes in AGs (*p* = 0.008). Pre‐GAHT and post‐GAHT average AGs of the runners did not differ with or without adjustment (*p* = 0.304) for training differences. Average race times for the swimmer increased by 5.2 ± 2.3% and FINA score increased by 65 points after 34 months of GAHT. In our sample of TW athletes taking GAHT, longer distance events were associated with larger decrements in performance compared with shorter distance events, with exercise training helping attenuate these decrements. Event demands and exercise training may be important considerations in understanding the effects of GAHT on athletic performance in TW athletes.

## Highlights

- This is the first study in TW athletes to use both retrospective and prospective methods to assess decreases in athletic performance, changes in training and hormone concentrations both pre‐GAHT and post‐GAHT.
- Nine TW runners had decreases in performance of approximately 15% with > 1 year of GAHT whereas 1 TW swimmer had decreases in performance of approximately 5%.
- In both running and swimming, the performance decrements were greater in longer‐duration (> 240 s) events than in shorter‐duartion (< 120 s) events.
- Using sport specific evaluation methods, the TW runners were collectively equally as competitive in the men's category pre‐GAHT and the women's category post‐GAHT, whereas the swimmer was more competitive post‐GAHT in the women's category than in the men's category pre‐GAHT.

## 1 Introduction

Most sports are segregated by sex (Ljungqvist 2018) primarily due to the known differences in endurance, strength and athletic performance between men and women (Hunter et al. 2023). The sex‐based differences in performance are associated with the ∼10–20‐fold higher circulating testosterone levels in males than females after the onset of puberty (Handelsman et al. 2018; Handelsman 2017). This androgenisation results in males having higher stature (Gray and Wolfe 1980), greater lean body mass (Suetta et al. 2019), higher blood haemoglobin concentration (Otto et al. 2013) and larger heart and lung size (St Pierre et al. 2022) than females. Consequently, males demonstrate ∼10% athletic superiority over females in endurance sports, such as swimming and running (Sandbakk et al. 2018), and up to 60% in sports requiring high levels of upper body strength, such as powerlifting (Keogh et al. 2008).

Transgender individuals have a gender identity that differs from their natal sex and often take gender affirming hormone therapy (GAHT) to align their body and identity (Coleman et al. 2012). Oestrogen with a medication to suppress or block testosterone is typically given as GAHT for TW (male natal sex and female gender identity) (Seal 2016). In nonathletic transgender adults, systematic and narrative reviews have summarised the effects of GAHT on physiological measures (Harper et al. 2021; Hilton and Lundberg 2021). Specifically, haemoglobin concentration [important for endurance sports (Mairbäurl 2013)] is reduced after 4 months of GAHT, with TW demonstrating female typical levels (Wiik et al. 2020). In contrast, after 12–36 months of GAHT, despite significant decreases in muscle cross‐sectional area in nontrained TW, lean body mass and strength remained higher than observed in cisgender women (female natal sex and gender identity; CW) (Harper et al. 2021; Hilton and Lundberg 2021).

Few studies have documented long‐term changes in athletic performance in transgender athletes. Two peer‐reviewed studies have analysed performance changes in trans athletes post‐GAHT (Harper 2015; Senefeld et al. 2023). The first study (Harper 2015) was a retrospective examination of self‐reported performance times from 8 TW competing in long‐distance running. Collectively, the performance times were 17% slower after 1–20 years of GAHT. This percentage is consistent with the difference in endurance running performance reported between elite level male and female athletes (10%–13%) (Cheuvront et al. 2005) once the loss of speed associated with ageing is accounted for. However, this study (Harper 2015) lacked rigour due to several methodological weaknesses: only ∼50% of the self‐reported times were verified, training differences pre‐GAHT and post‐GAHT were not reported and testosterone, oestrogen and haemoglobin concentrations were not measured. The second study (Senefeld et al. 2023), a more recent case study of a TW swimmer, reported that performance slowed by ∼5% after 2 years of GAHT. However, the participant was subsequently more successful in national collegiate athletic association (NCAA) competition in the female category post‐GAHT than she had been in the male category pre‐GAHT. In addition to the 2 retrospective studies of field performance in trans athletes, a cross‐sectional laboratory study examined hand‐grip strength, countermovement jump (CMJ) and V̇O_2peak_ in 23 TW, 21 CW, 12 transgender men (TM) and 19 cisgender men (CM) (Hamilton et al. 2024) who were all athletically trained. The TW possessed higher absolute hand‐grip strength and lower relative V̇O_2max_ than the CW, whereas there was no significant difference in the CMJ height, relative hand‐grip strength and absolute V̇O_2max_ between the 2 groups (Hamilton et al. 2024). Another cross‐sectional study examined 7 TW, 8 CW and 8 CM volleyball players for parameters including handgrip strength, CMJ and V̇O_2peak_ (Alvares et al. 2025). The TW possessed similar handgrip strength, CMJ height and absolute and relative V̇O_2peak_ to the CW but spent significantly less time practising volleyball (4 h·wk^−1^ vs. 14 h·wk^−1^) than the CW (Alvares et al. 2025). The data from the 4 studies suggest that trans and cis female athletes may perform similarly in some but not all measures.

The dearth of longitudinal pre‐GAHT and post‐GAHT data on trans athletes has limited the understanding of the performance effects of GAHT in TW athletes and limited the ability of sports‐governing bodies to make informed policy around transgender athlete inclusion. Consequently, the aims of this study were to collect and analyse verified, self‐reported retrospective and prospective information on competition results, training and hormonal data from TW competing in swimming and running pre‐GAHT and post‐GAHT initiation. To provide further context, we also evaluated these results using sport‐specific scoring systems, namely FINA scores for swimmers and age‐grade scores (AGs) for runners.

## 2 Materials and Methods

### 2.1 Participants

Participants were recruited online by accessing virtual groups supportive of transgender athletes or by direct email communication between the lead author and known TW athletes. The following inclusion criteria were applied: competing in running or swimming pre‐GAHT and post‐GAHT, age 18–50 years, healthy and able to participate in exercise and have publicly available competition results. Exclusion criteria were known cardiovascular or metabolic disease and somatic or psychotic disorders. All experimental procedures were approved by the Loughborough University Ethics Sub‐Committee (approval number 2020‐1631–1726) and conformed to the Declaration of Helsinki, except for registration in a database. All participants signed an informed consent document prior to data collection. Participants provided links to publicly available competition results websites and the lead author verified all results. An independent search for competition results was also undertaken, with relevant results added to the database. A total of 10 TW (9 runners and 1 swimmer) submitted retrospective performance data at study enrolment and continued to prospectively self‐report performances for 12 months after study enrolment.

### 2.2 Groupings

Given that there are different physiological parameters of importance in aerobic and anaerobic performance (Mairbäurl 2013; Barbieri et al. 2017), and that swimmers cover less distance per second than runners, time duration was chosen to differentiate between long and short events when the two sports were combined. Performance changes with GAHT in running and swimming were determined for short (< 120 s) and long (> 240 s) duration events. There were also two groupings chosen to compare pre‐GAHT and post‐GAHT performance. One group used all pre‐GAHT and post‐GAHT performance times to maximise the number of data points. A second grouping was analysed using only data points within ± 3 years of GAHT (GAHT‐3) initiation to minimise the effect of time varying confounders (Mansournia et al. 2017).

### 2.3 Self‐Reported Blood Tests and GAHT Data

The participants self‐reported current and past blood tests ordered by their physician for testosterone, oestrogen, haemoglobin and haematocrit. If necessary, results were converted into the international system of units (SI); nmol·L^−1^ for testosterone, pmol·L^−1^ for oestrogen, g·L^−1^ for haemoglobin and % for haematocrit using recognised conversion calculations (Rxph 2023). The participants also reported the drug combinations within their GAHT regimen. No medications were given as a part of this study. Participants also reported height and body mass pre‐GAHT and post‐GAHT. Body mass index (BMI; kg·m^−2^) was then calculated.

### 2.4 Runners Age Grade Athletics Scores

Runners' race times were converted into age‐grade scores, by expressing performance relative to the best performance achieved by a runner of the same age and sex using Equation (1). (Grubb 1998):

(1) AG=(ASx100)/RT,

where AG is the age grade score, AS is the best performance of a runner in s of the same age and sex and RT is the performance of the runner in the study in s. AGs are routinely used by World Masters Athletics to evaluate performances (Medic 2012). The pre‐GAHT AGs for the TW in the study were calculated using male standards whereas the post‐GAHT AGs were calculated using female standards.

### 2.5 Swimming FINA Scores

The swimmer's times were converted to FINA scores (Więckowski and Kołodziejczyk 2020) using Equation (2). (Aquatics 2023):

(2) $$P=1000\;*\;{\left(B/T\right)}^{3},$$

where *P* is the FINA score, *T* is swimmer's time in s and *B* is the base time representing the existing world record for the swimmer's sex in s. (Aquatics 2023) FINA scores are routinely listed in swimming results (Swimcloud 2024).

FINA scores were calculated for the TW swimmer pre‐GAHT using male records and post‐GAHT using female records. FINA scores were also captured from public records on women's freestyle events in both the 2019 and 2022 NCAA division 1 (D1) swimming championships.

### 2.6 Training Volume

Upon study entry, and every 3 months thereafter, the participants submitted a report detailing the frequency of training sessions (i.e., the number of training sessions per week) and session duration (i.e., the length in min of the sessions) completed in a typical week. There was a single training report for the pre‐GAHT timeframe and one report for each timeframe after study entry. The training report included running or swimming training as well as strength training.

### 2.7 Data Handling and Statistical Methods

A set of single best performance times pre‐GAHT and at least 5 months post‐GAHT for each participant in each event was compiled. Changes in performance pre‐GAHT versus post‐GAHT were calculated as percentage change. The percentage performance decrements with GAHT for short (< 120 s) and long (> 240 s) duration events were compared using an independent *t*‐test. After normal data distribution was determined, the running performances pre‐GAHT and post‐GAHT were compared using Student's *t*‐tests. Mean ± standard deviation (SD) and 95% confidence interval (CI) are reported, with statistical significance set at *p* < 0.05. Cohen's *d* was calculated as the ratio of the time difference to the pooled standard deviations and we considered *d* = 0.2 a small effect, *d* = 0.5 a medium effect and *d* = 0.8 a large effect (Goulet‐Pelletier and Cousineau 2018). AGs were calculated for the entire runner cohort, and separately for sprints (60–400 m), middle‐distance (800–3219 m) and long distance (≥ 5000 m) events.

Two models were built to analyse mean AG controlling for training. In the first, the mean AG and percentage change in training was calculated for each runner in both pre‐GAHT and post‐GAHT conditions across all events and linear regression was used to fit the data. In a second model, mean AG was calculated for each participant at each training volume for each category of event. Where the participant had AGs both pre‐GAHT and post‐GAHT for a given category, the difference in means for that category and percentage training change were calculated and an ANCOVA (with training volume as the covariate) was used to determine the effect of training and category on the change in mean AG. Graph Pad (Graph Pad Prism 9.5.1, GraphPad Boston, MA), R (base version 4.2.2) and SPSS (28.0.1.1, SPSS Inc., Chicago, IL) were used for data analysis.

## 3 Results

### 3.1 Participant Characteristics

Participant characteristics for TW (*n* = 10) at study entry are shown in Table 1, whereas pre‐GAHT and post‐GAHT height and body mass are shown in Table 2. Nine TW runners and 1 TW swimmer were 34.7 ± 9.8 years old. GAHT duration at study entry was 44.1 ± 40.4 months, with a variety of physician prescribed GAHT regimens reported. Ten TW reported greater pre‐GAHT than post‐GAHT height (pre vs. post, 1.78 ± 0.07 m vs. 1.77 ± 0.07 m; *p* = 0.039; 95% CI = −0.027,−0.0009; *d* = 0.2) but no pre‐to post‐GAHT difference (*p* > 0.2 all and *d* < 0.3; all) in body mass (71.9 ± 9.6 kg–72.5 ± 8.9 kg) or body mass index (22.7 ± 2.9 kg·m^−2^–23.3 ± 3.1 kg·m^−2^) (Supporting Information S1: Table 1).

**TABLE 1:** Participant characteristics at study entry.

I.D. number	Age (years)	Sport	GAHT regimen	Gonadectomy	GAHT duration (months)
1	42	Sprinting	OE + P	Yes	108
2	44	Sprinting	OE	Yes	99
3	47	Sprinting	CA + EG	No	23
4	23	MD running	spiro + OE + P	No	21
5	41	LD running	spiro + OE	No	2
6	48	LD running	spiro + OE	No	33
7	28	LD running	IE	Yes	38
8	32	LD running	spiro + IE + P	No	25
9	35	LD running	spiro + OE	No	10
10	22	Swimming	spiro + OE + P	No	32

Abbreviations: CA = cyproterone acetate; EG = oestrogen gel; IE = injectable oestrogen; LD = long‐distance (≥ 5000 m); MD = middle‐distance (800–3219 m); OE = oral oestrogen; P = progesterone; spiro = spironolactone; Sprints = (60–400 m).

**TABLE 2:** Self‐reported, pre‐GAHT and post‐GAHT height, body mass, and body mass index (BMI) of 10 trans women.

Participant number	Pre‐GAHT			Post‐GAHT		
	Height (m)	Body mass (kg)	BMI (kg·m^−2^)	Height (m)	Body mass (kg)	BMI (kg·m^−2^)
1	1.69	85	29.8	1.69	90	31.5
2	1.85	83	24.3	1.81	75	22.9
3	1.82	78	23.5	1.82	80	24.2
4	1.83	69.4	20.7	1.83	71	21.3
5	1.73	60	20	1.73	63	21
6	1.78	65	20.5	1.78	74	23.4
7	1.75	66	21.6	1.73	64	21.2
8	1.68	63.5	22.5	1.67	63.5	22.8
9	1.78	65	20.5	1.73	65	21.7
10	1.90	83.9	23.2	1.88	79	22.5
Mean ± SD	1.78 ± 0.1	71.9 ± 9.6	22.7 ± 2.9	1.77 ± 0.1	72.5 ± 8.9	23.3 ± 3.1

Abbreviation: GAHT = gender‐affirming hormone therapy.

### 3.2 Blood Measures

There were significant (*p* < 0.01 and *d* > 0.8; all) changes in pre‐GAHT and post‐GAHT testosterone [*n* = 9 (including the swimmer)], oestradiol (*n* = 8), haemoglobin concentrations (*n* = 4) and haematocrit (*n* = 4) (Table 3). Female‐typical testosterone concentrations (< 1.8 nmol·L^−1^) for the TW were observed after 5 ± 4 months of GAHT.

**TABLE 3:** Mean pre‐GAHT and post‐GAHT blood measures in trans women participants.

Measure	*n*	Pre‐GAHT	No. days pre‐GAHT	Post‐GAHT	No. months post‐GAHT	*p*	Cohen's *d*
T (nmol/L)	9	18.4 ± 2.8	33 ± 18	1.10 ± 0.52	15 ± 09	< 0.001	8.6
Hgb (g/L)	4	153 ± 13	17 ± 21	135 ± 13	29 ± 19	< 0.001	1.4
HCT (%)	4	43.2 ± 4.0	17 ± 21	40.0 ± 4.0	29 ± 19	< 0.001	0.8
E (pmol/L)	8	103 ± 62	43 ± 78	673 ± 386	19 ± 14	0.006	2.1

Abbreviations: E = oestradiol; GAHT = gender‐affirming hormone therapy; HCT = haematocrit; Hgb = haemoglobin; T = testosterone.

**FIGURE 1:**
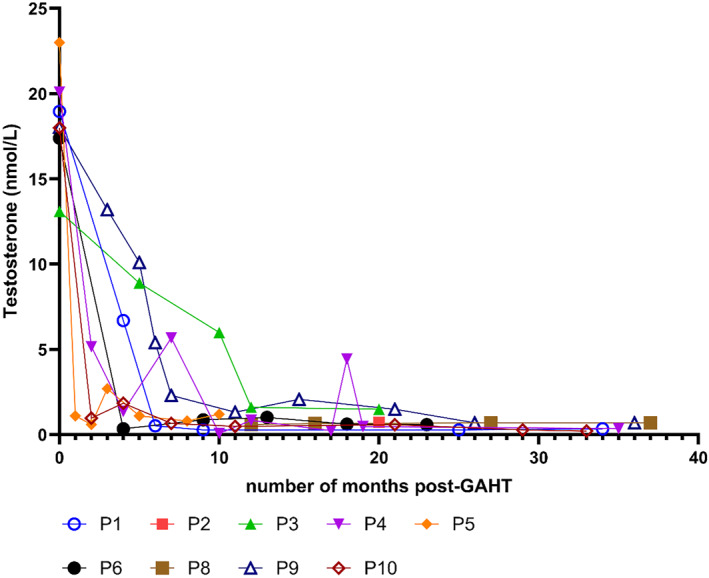
Testosterone concentrations in 9 TW with GAHT duration. P = participant.

Testosterone concentrations of 9 TW (including the swimmer) are displayed in Figure 1. The pre‐GAHT testosterone concentrations are assigned to 0 months.

### 3.3 Best Pre‐GAHT and Post‐GAHT Performances

The best pre‐GAHT performances were identified for the TW runners and TW swimmer at 48 ± 53 months (range: 2–143 months) and 2–27 months pre‐GAHT initiation, respectively. The best post‐GAHT performances for the TW runners and TW swimmer were identified at 31 ± 23 months (range: 5–86 months) and 31–34 months post‐GAHT initiation, respectively (Tables 4 and 5).

**TABLE 4:** Best performance times for 9 TW runners pre‐GAHT and post‐GAHT.

ID no.	Distance	Pre race time (h:m:s)	Pre duration (mos)	Pre age (yrs)	Post race time (h:m:s)	Post duration (mos)	Post age (yrs)	Time increase (%)
3*	60 m	8.03	10	45	8.56	24	47	6.6
2	60 m	7.44	6	35	8.10	78	42	8.9
3*	100 m	12.59	7	45	13.18	17	47	4.7
1	100 m	11.73	143	18	13.54	86	37	15.4
2	100 m	10.83	87	28	12.50	44	39	15.4
3*	200 m	24.94	9	45	26.96	19	47	8.1
1	200 m	23.7	118	20	28.14	86	37	18.7
2	200 m	21.47	97	28	25.22	44	39	17.5
3*	400 m	56.67	6	45	59.77	30	48	5.5
4*	1 mile	04:09.3	2	20	04:45.8	20	22	14.6
6*	1 mile	04:55.0	22	44	05:26.0	28	47	10.5
4*	3000 m	08:42.3	4	20	09:59.8	20	22	14.8
9*	2 miles	10:25.0	6	34	12:20.0	12	35	18.4
9*	5 km	16:33.0	14	33	19:11.0	9	35	15.9
6*	5 km	16:46.0	10	45	18:48.0	25	47	12.1
5*	5 km	17:12.0	30	39	20:25.0	11	42	18.7
8	5 km	15:03.0	118	20	17:28.0	29	32	16.1
8	8 km	24:47.0	124	19	31:15.0	25	32	25.4
9*	5.35 miles	30:51.0	3	34	38:24.0	14	35	24.5
5	10 km	33:40.0	65	36	39:40.0	5	42	17.8
8	10 km	31:21.0	131	19	38:47.0	23	32	23.7
6*	Half mar	01:17:49	9	45	01:26:16	26	47	10.9
8	Half mar	01:11:08	121	20	01:21:43	31	32	14.9
7*	Marathon	02:48:58	5	24	03:08:50	13	26	11.8

Abbreviations: 1 mile = 1609 m; half mar = half marathon (21.1 km); km = kilometres; m = metres; marathon = 42.2 km; Pre (Post) age = age in years at which pre (post) GAHT races occurred; Pre (Post) dur = number of months pre (post) GAHT initiation; Pre (Post) Dur. mos = duration in months pre (post) GAHT * = paired times within 3 years of GAHT initiation; Pre (post) race time = pre (post) GAHT race time in sec (s) or min sec (m:s) or hrs min sec (h:m:s).

**TABLE 5:** Race times for 1 transgender woman swimmer pre‐GAHT and post‐GAHT.

Distance (yards)	Pre time (m:s)	Age (yrs)	Months pre‐GAHT	FINA score	NCAA rank	Post‐time (m:s)	Age (yrs)	Months post‐GAHT	Time increase (%)	FINA score	NCAA rank
100	47.15	17	27	N/A	N/A	47.37	22	34	0.5	890	13
200	01:39.3	19	5	732	552	01:41.9	22	31	2.6	919	3
500	04:18.7	19	2	873	65	04:33.2	22	34	5.6	903	1
1650	14:54.8	19	2	883	32	15:59.7	22	31	7.3	862	13

*Note:* FINA score and NCAA rank are not applicable for the pre‐GAHT 100‐yard race as it was swum in a high school relay.

Abbreviations: Pre (post) race time = pre (post) GAHT time in min sec (m:s); Pre (Post) GAHT (mos) = the number of months pre (post) GAHT at which race occurred; Yrs = years.

### 3.4 Runners' Performance and Age Grade Athletics Scores

From 9 runners, 208 verified self‐reported and 41 independently gathered race performances were collected. Eight TW competed in more than 1 event, with 3 competing in sprint events (60–400 m), 3 competing in middle‐distance events (800–3219 m) and 5 competing in long‐distance events (≥ 5000 m). From the 249 performance times, 24 pairs of the best pre‐GAHT and post‐GAHT times for 12 different athletic running events were identified and evaluated (see Table 4).

Post‐GAHT times over distances from 60 m to the marathon increased from pre‐GAHT times [*p* = 0.002, *d* = 0.72 and 95% CI of difference (84,326)], with an overall increase of 14.6 ± 5.6%. Times increased by 11.2 ± 5.5% in sprint events, 14.6 ± 3.2% in middle‐distance events and 17.4 ± 5.2% in long‐distance events.

The fastest pre‐GAHT times were observed at age 31.7 ± 10.6 years (range: 19–45 years) and the fastest post‐GAHT times were observed at 38.1 ± 8.2 years (range: 22–49 years).

**FIGURE 2:**
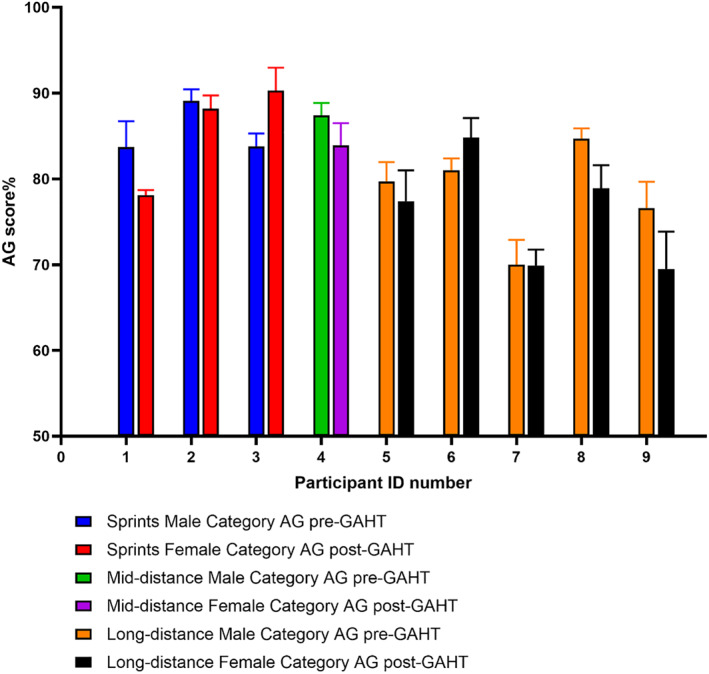
Age grade scores from 9 TW runners. Error bars represent standard deviation. 100% AG = best time for age and sex. Higher AGs = better performance.

All 249 times were used to calculate AGs for the 9 TW runners (Figure 2).

There was no difference in the overall average AGs [*p* = 0.30, *d* = 0.38 and 95% CI of difference (−5.2,1.8)] of the cohort pre‐GAHT and post‐GAHT (83.1 ± 4.9% pre‐GAHT and 81.6 ± 7.5% post‐GAHT). Pre‐GAHT and post‐GAHT average AGs were 86.8 ± 3.0% and 88.1 ± 4.6% for the sprints [*p* = 0.20, 95% CI of difference (−0.7, 3.3)], 86.6 ± 3.5% and 81.0 ± 7.5% for the middle‐distance [*p* = 0.005, 95% CI of difference (−9.2, −1.9)] and 79.7 ± 3.7% and 77.0 ± 5.5% for the long‐distance events [*p* = 0.006, 95% CI of difference (−4.7, −0.8)]. Pre‐GAHT and post‐GAHT average AGs calculated from the 24 pairs of best times were 83.1 ± 4.9% and 82.8 ± 7.4% [*p* = 0.77, 95% CI of difference (−2.5, 1.8)].

### 3.5 Training Practices of Runners

**FIGURE 3:**
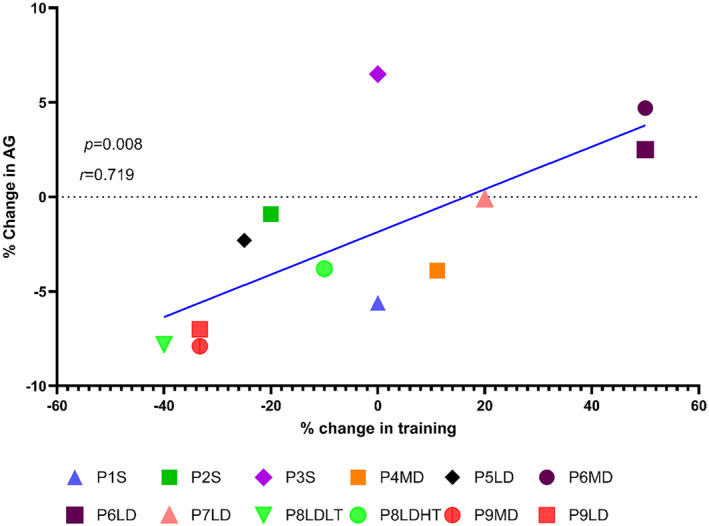
Pre‐GAHTto post‐GAHT % change in AG score with % change in self‐reported training volume in 9 TW runners. HT = higher training volume; LD = long‐distance; LT = lower training volume; MD = middle‐distance; P = participant; S = sprint.

The runners reported 560 ± 160 min·week^−1^ of running specific training sessions pre‐GAHT and 536 ± 149 min·week^−1^ post‐GAHT (Supporting Information S1: table 2), a nonsignificant difference [*p* = 0.60, *d* = 0.2 and 95% CI of difference (−129,80)]. The percentage change in the number of training min·week^−1^ in individual participants ranged from −40% to + 50%. The change in AGs was −1.5 ± 0.8%. Two runners ran both middle‐ and long‐distance races. One runner (ID = 8) had markedly different post‐GAHT training volumes (300 and 450 miṅ·week^−1^) at different periods in the prospective study period and ran faster when she was training more. Thus, Figure 3 contains 12 points for 9 runners. Only 2 runners reported strength training both pre‐GAHT and post‐GAHT (Supporting Information S1: Table 2).

There was a positive strong correlation (*r* = 0.719 and *p* = 0.008) between the pre‐to post‐GAHT changes in AGs versus training (see Figure 3), and the resulting linear regression equation was ∆ AGs = 0.113 ∆training −1.85 [95% CI on the regression line slope = (0.036, 0.190)]. Using the ANCOVA, the resulting training‐adjusted pre‐to post‐GAHT AG differences were 0.9% [95% CI (−3.0 to 4.8)], −1.4% [95% CI (−3.7 to 0.9)] and −0.8% [95% CI (−3.1 to 1.5)] for the sprints, middle‐ and long‐distance races, respectively. The coefficient of percentage change in training was 0.12 [95% CI (0.042, 0.20)].

### 3.6 Swimming Performance and FINA Scores

The TW swimmer competed pre‐GAHT and post‐GAHT in NCAA freestyle events, which included the 200, 500 and 1650 yards. Training volume was reported at 1080 min·week^−1^ both before and after 31–34 months of GAHT. The swimmer also competed in the 100‐yard freestyle event in the NCAA post‐GAHT and as a 17‐year‐old high school student (pre‐GAHT). Her training volume in high school is unknown. Pre‐GAHT and post‐GAHT swim race times, complete with FINA scores and NCAA rankings (except for the high school race) for the swimmer, are presented in Table 5.

**FIGURE 4:**
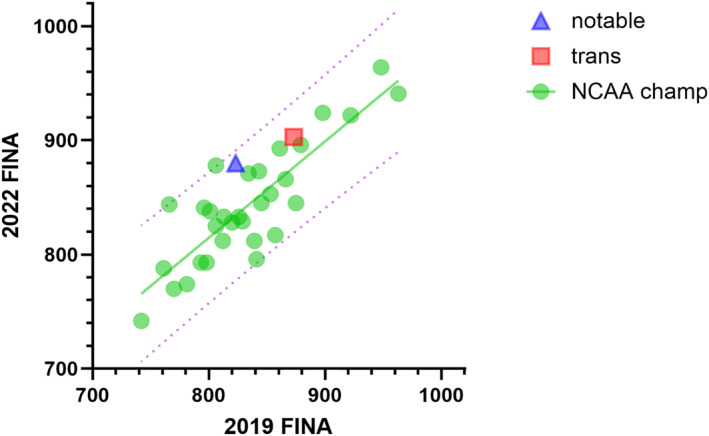
FINA scores from freestyle swims in the Women's 2019 and 2022 NCAA D1 championships. The solid line is the linear regression whereas the dotted lines denote the 95% prediction values.

Pre‐to post‐GAHT FINA scores increased by 187 and 30 points in the 200‐ and 500‐yard events, respectively, and decreased by 21 points in the 1650‐yard event. The FINA scores from competitors who swam the same freestyle event (50, 100, 200, 500 or 1650 yd) in both the 2019‐ and 2022‐Women's NCAA D1 championship are displayed in Figure 4. The 500‐yard freestyle FINA scores from the trans swimmer and the 200‐yard freestyle FINA scores from a notable swimmer achieved in their 2019 conference championships and the 2022 Women's NCAA D1 championships (where the two swimmers tied in the 200‐yard freestyle) are also displayed. Points above the regression line denote improvement beyond the mean (9.5 ± 28.2) increase, including the trans and the notable swimmers.

### 3.7 GAHT‐3 Data

Using only GAHT‐3 data, 14 pairs of best times were included for the runners (indicated by * in Table 4 or in Supporting Information S1: Table 3). All the swimmer's times fell into the GAHT‐3 range (paired results in Table 5). Overall, the best pre‐GAHT‐3 performances were identified at 9 ± 8 months (range: 2–30 months) and the best post‐GAHT performances were identified at 21 ± 8 months (range 9–34 months). In the runners, average pre‐to post‐GAHT‐3 times increased by 12.7 ± 5.6% [*p* = 0.034, *d* = 0.63, 95% CI of difference (87,395)], with time increases of 6.2 ± 1.5% in sprint events, 14.6 ± 3.2% in middle‐distance events and 15.7 ± 5.2% in long‐distance events.

### 3.8 Performance Decrements in Shorter Versus Longer‐Duration Events

Using data from the entire cohort, and the GAHT‐3 data, the percentage race time increases in swimming and running were larger for longer races (> 240 s duration) than in shorter races (< 120 s duration). When the entire cohort was used, the differences were 15.8 ± 5.6 versus 10.3 ± 5.9%, respectively; *p* = 0.038, 95% CI of difference (0.3–10.0). When the GAHT‐3 data were used, the differences were 13.8 ± 5.2 versus 5.5 ± 2.1%, respectively; *p* < 0.001, 95% CI of difference (4.5–12.0).

## 4 Discussion

In a cohort of TW runners, GAHT was associated with an overall ∼15% increase in race times whereas GAHT‐3 was associated with an overall ∼13% increase in race times, with middle‐ and long‐distance running events demonstrating greater decrements in performance compared with sprint events. Changes in training volume were positively and significantly associated with changes in AGs. Training‐adjusted and unadjusted AGs in the entire cohort of runners, both before and after GAHT, did not differ. In the TW swimmer, overall race times were increased to a lesser extent (∼5%) after 31–34 months of GAHT, with larger performance decrements also observed for longer versus shorter distance events. Accordingly, FINA swim scores were decreased and increased, respectively. Although the exact effect of GAHT on athletic performance in TW athletes was not able to be determined, these findings suggest that GAHT impairs athletic performance in TW runners and swimmers, with greater decrements observed in longer versus shorter distance events. In TW runners, exercise training may help attenuate these performance decrements.

### 4.1 Trans Women Runners

The increase in performance times in the 9 TW runners varied substantially across participants, ranging between 6%–24%, with an overall average increase of 14.6%. The magnitude of this increase also varied by event specialism, with greater increases observed in middle‐ (14.6%) and long‐distance events (17.4%) compared to sprint events (11.2%). Reasons for the larger performance decrements in longer versus shorter distance events in TW runners is unclear. However, previous studies (Olson‐Kennedy et al. 2018; Defreyne et al. 2018; Vita et al. 2018) and a systematic review (Harper et al. 2021) have identified that haemoglobin and haematocrit in nonathletic TW on GAHT decrease relatively quickly from male to female values. In contrast, hand grip and knee extension strength, lean body mass and muscle cross‐sectional area have been demonstrated to show a slower rate of decline, with nonathletic TW remaining stronger than nonathletic CW after 36 months of GAHT (Harper et al. 2021; Hilton and Lundberg 2021; Klaver et al. 2018; Scharff et al. 2019; Tack et al. 2018; Van Caenegem et al. 2015). Haemoglobin is a key determinant of endurance performance (Mairbäurl 2013), whereas lean body mass and strength contribute to sprint performance to a large extent (Barbieri et al. 2017). Thus, the greater change in performance times in middle‐ and long‐distance runners may be associated in part with the rapid GAHT‐mediated decrease in haemoglobin mass (Olson‐Kennedy et al. 2018; Defreyne et al. 2018; Vita et al. 2018). Conversely, the smaller decrement in sprint versus endurance performance in TW runners may be explained in part by the slower rate of decline in lean body mass and strength (Harper et al. 2021; Hilton and Lundberg 2021; Klaver et al. 2018; Scharff et al. 2019; Tack et al. 2018; Van Caenegem et al. 2015). Although not able to be confirmed in the current study, it is plausible that in athletes, in whom resistance training is implemented to increase muscular strength, power and muscle size, the GAHT‐associated rate of decline in both lean mass and strength in TW may be attenuated. Studies to examine this postulate are needed.

In runners, comparison of all performance times versus those using the GAHT‐3 grouping demonstrated marginal changes in average race times, shifting from ∼15% to ∼13% overall, and from ∼17 to ∼16% for long‐distance events, with middle‐distance events remaining at ∼15%. In contrast, changes in race times for the sprints shifted from ∼11% to ∼6%. However, the number of eligible sprinters fell from 3 to 1 when the GAHT timeframe was restricted. Further studies are needed to determine a more accurate estimate of the performance changes with shorter versus longer GAHT duration.

The ∼15% average increase in performance time in the entire cohort of runners after 31 ± 23 months of GAHT is marginally larger than the 10%–13% average difference previously reported between elite male and female runners at distances between 100 m and the marathon (Cheuvront et al. 2005). In the current study, the runners were ∼38 years old at the time of their best post‐GAHT races compared with ∼32 years of age at the time of their best pre‐GAHT races. The ability to run fast starts to diminish at ∼30 years of age largely due to age‐related reductions in muscle size and force‐generating capacity of the lower limb muscles, which results in stride length reduction, increased ground contact time (Korhonen et al. 2009) and deteriorations in V̇O_2max_, lactate threshold and running economy (Tanaka and Seals 2008). Whether the older age of our TW runners in is capturing a window of increasing age‐associated decline in performance per se could not be determined. However, AGs were used to allow for comparison of runner's performance times while accounting for the loss of speed due to ageing (Grubb 1998). Although the independent effects of GAHT on AGs cannot be determined, the AGs normalise race times to world record speed for an age and sex matched athlete at a given distance. Before GAHT, average AGs were 83.1 ± 4.9%. Scores in this range typically reflect regional to national level runners (Grubb 1998). The average pre‐GAHT and post‐GAHT AGs were not statistically different for the entire cohort of runners, or for sprinters, but were decreased for middle‐ and long‐distance runners. However, after adjustment for self‐reported training exposure, AGs did not differ for any category, suggesting that, after 5–86 months of GAHT, this cohort of runners were approximately as competitive relative to their age‐group in the women's category as they had been in the men's category competing against age‐matched males pre‐GAHT. Whether similar or different, findings would be observed in TW runners of a different age competing at different levels of competition remain to be determined. However, we observed a strong positive correlation between AGs and the self‐reported training volume. Thus, increased exercise training volume may help attenuate GAHT associated decrements in performance and/or improve AGs in TW runners.

### 4.2 Trans Woman Swimmer

Given that the swimmer's last pre‐GAHT 100‐yard race occurred as a 17‐year‐old high school student, it is not reasonable to make a direct comparison with her post‐GAHT 100‐yard races as a 22‐year‐old. The increase in performance times in the swimmer varied substantially across other events, ranging from 2.6% in the 200‐yard race to 7.3% in the 1650‐yard race. It is unclear if the smaller percentage increase in the swimmers' performance compared with the runners reflects a potential sport‐specific or event‐duration effect.

The swimmer's 500‐yard 34‐month post‐GAHT FINA score (903) in the women's category was 30 points larger than her highest pre‐GAHT FINA score (873) in the men's category. In a study of 20 swimmers not identified by sex/gender (Więckowski and Kołodziejczyk 2020), 7 demonstrated increased single best FINA scores of 33–60 (42 ± 9) FINA points over one season. The 30‐point increase in FINA score of the TW between the 2018 and 2019 and between 2021 and 2022 seasons was compared to NCAA D1 championship‐level female freestyle swimmers over the same timeframe. This 30‐point increase was greater than but within one standard deviation of the mean increase of 9.5 FINA points (Figure 3). In contrast, the swimmer's 500‐yard performance resulted in an improvement in NCAA D1 ranking, from 65^th^ to first (out of approximately 1500 NCAA D1 swimmers), raising the question of the most relevant measure of performance, and how to best compare CW and TW swimmers.

### 4.3 Limitations

Although the data presented are novel, the authors acknowledge several limitations. The participants were highly heterogeneous, including a wide age range and duration of GAHT exposure. Participants also competed in different events and at different performance standards. Many of the data were gathered retrospectively. The self‐reported training data could not be verified, and the duration of training sessions did not necessarily match the duration of running or swimming. Additionally, the reported strength training was too inconsistent to draw any conclusions. The hormone concentration data came from multiple labs, which may have employed different methods of data analysis. The study was not able to control for duration of GAHT or for differing GAHT regimens, making it difficult to determine the role of GAHT on performance. There was no control over pre‐GAHT performance timing with respect to GAHT initiation. Other factors that could affect performance, such as injuries, motivation, mental health status and coaching, were not collected and thus not able to be controlled. Only two sports (athletics and swimming) are represented in this study, and the sample sizes were small, with 9 runners and 1 swimmer. The anthropomorphic data were self‐reported, but the accuracy of the measurements is unknown. Although the pre‐to post‐GAHT difference in height was statistically significant, it was of trivial athletic importance. The measurement error in height may have been larger than the reported pre‐to post‐GAHT difference. It is also unlikely that the self‐reported height loss of 4 and 5 cm in participants 4 and 9 was accurate. Taking these limitations into consideration, these data should be interpreted accordingly.

## 5 Conclusions

The current study retrospectively and prospectively investigated the effects of GAHT on athletic performance in a small cohort of TW athletes. In the TW runners, overall race times increased ∼15% after 5–85 months of GAHT. Changes in training volume were positively associated with changes in AG scores. Training‐adjusted and unadjusted AGs before and after GAHT did not differ in the entire runner cohort. In the TW swimmer, average race times increased to a lesser degree than in runners (∼5%) after 31–34 months of GAHT. Pre‐GAHT versus post‐GAHT FINA scores were increased in shorter and decreased with longer distance events. In all TW athletes, greater decrements in performance were observed in longer versus shorter duration events. Although the exact role of GAHT on athletic performance in TW athletes was not able to be determined, these findings suggest that shorter duration events necessitating greater muscular strength and power may be associated with smaller decrements in performance than longer duration events demanding greater muscular endurance and aerobic power. Moreover, in TW runners, exercise training may help attenuate these GAHT‐associated losses. Thus, the performance effects of GAHT in TW athletes may depend in part on the physiological demands of a given event and exercise training volume. Further studies to confirm these findings are needed.

## Author Contributions

J.H., E.O.D. and G.L.W. devised the study. J.H. gathered the data. J.H., E.H., R.C.B. and E.O.D. completed the data analysis. All authors have contributed to the manuscript. E.O.D. is the guarantor.

## Conflicts of Interest

The authors declare no conflicts of interest.

## Supporting information


Supporting Information S1

## Data Availability

Anonymised study data are available upon request.
