# Supplementary material for: Longitudinal Performance Changes in Transgender Women Athletes Pre and Post Gender Affirming Hormone Therapy
Source: Eur J Sport Sci. 2025 Aug 13;25(9):e70036. doi: 10.1002/ejsc.70036 (PMC12350078; doi:10.1002/ejsc.70036)
Supplement: Supplementary file 1 — Supporting Information S1 [file EJSC-25-e70036-s001.docx]

**Supplemental Data**

**Height, Body Mass, and Body Mass Index data**

The participants were asked to self-report height (m) and body mass (kg) for each time point that accompanied a performance record. The 10 trans women reported greater pre- than post-GAHT height (pre vs post, 1.78 ± 0.07 m vs 1.77 ± 0.07 m; *p*= 0.039; 95% CI = -0.027,-0.0009; *d*=0.2, small) but no pre- to post-GAHT difference (*p*>0.2 all, d<0.3 all, small) in body mass (71.9 ± 9.6 kg to 72.5 ± 8.9 kg) or body mass index (22.7 ± 2.9 kg∙m^-2^ to 23.3 ± 3.1 kg∙m^-2^) (supplemental table 1)

**Supplemental Table 1**. Self-reported, pre-, and post-GAHT height, body mass, and body mass index (BMI) of 10 trans women.

|  | Pre GAHT | | |  | Post GAHT | | |
| --- | --- | --- | --- | --- | --- | --- | --- |
| Participant | Height | Body mass | BMI |  | Height | Body mass | BMI |
| number | (m) | (kg) | (kg∙m^-2^) |  | (m) | (kg) | (kg∙m^-2^) |
| 1 | 1.69 | 85 | 29.8 |  | 1.69 | 90 | 31.5 |
| 2 | 1.85 | 83 | 24.3 |  | 1.81 | 75 | 22.9 |
| 3 | 1.82 | 78 | 23.5 |  | 1.82 | 80 | 24.2 |
| 4 | 1.83 | 69.4 | 20.7 |  | 1.83 | 71 | 21.3 |
| 5 | 1.73 | 60 | 20 |  | 1.73 | 63 | 21 |
| 6 | 1.78 | 65 | 20.5 |  | 1.78 | 74 | 23.4 |
| 7 | 1.75 | 66 | 21.6 |  | 1.73 | 64 | 21.2 |
| 8 | 1.68 | 63.5 | 22.5 |  | 1.67 | 63.5 | 22.8 |
| 9 | 1.78 | 65 | 20.5 |  | 1.73 | 65 | 21.7 |
| 10 | 1.9 | 83.9 | 23.2 |  | 1.88 | 79 | 22.5 |
| Mean ± SD | 1.78 ± 0.1 | 71.9 ± 9.6 | 22.7 ± 2.9 |  | 1.77 ± 0.1 | 72.5 ± 8.9 | 23.3 ± 3.1 |

Although the pre- to post-GAHT self-reported height in the 10 trans women is statistically significant (*p* = 0.039) the difference (0.014 m, *d* = 0.2) is of trivial importance for athletic performance. These heights were self-reported to the nearest centimetre, but the accuracy of the measurements is unknown, and measurement error may have been larger than the reported pre- to post-GAHT difference. It is also unlikely that the self-reported height loss of 4 and 5 cm in participants 4 and 9 was accurate.

**Supplemental Table 2. Pre- and Post GAHT training volume**

| ____________________________________________________________ | | | | | |
| --- | --- | --- | --- | --- | --- |
| ID number | Sport | prim pre | prim post | St pre | St post |
| ____________________________________________________________ | | | | | |
| P1 | Sprinting | 420 | 420 | 0 | 0 |
| P2 | Sprinting | 900 | 720 | 240 | 120 |
| P3 | Sprinting | 720 | 720 | 0 | 240 |
| P4 | MD running | 540 | 600 | 150 | 0 |
| P5 | LD running | 440 | 330 | 0 | 0 |
| P6 | LD running | 420 | 630 | 0 | 0 |
| P7 | LD running | 500 | 600 | 120 | 120 |
| P8 | LD running | 500 | 300* | 0 | 100 |
| P9 | LD running | 600 | 400 | 0 | 0 |
| P10 | Swimming | 1080 | 1080 | 0 | 0 |
| ____________________________________________________________ | | | | | |

Training volume reported in min·wk^-1^. P = participant, MD = middle distance, LD = long distance, prim = primary training (running or swimming), St = strength training, pre = pre-GAHT, post = post-GAHT, participant 8 increased her training from 300 to 450 minutes per week during the study.

**Pre- and post-GAHT Performance Times Within 36 Months of GAHT Initiation**

A second examination of performance times from the 9 runners excluded pre- and post-GAHT times that were more than 36 months pre- or post-GAHT initiation. This resulted in 14 pairs of performance times from 6 runners (1 sprinter, 1 middle distance runner and 4 long-distance runners) (Supplemental table 3). The best pre-GAHT performances were identified for the participants at 9±8 months (range: 2-30 months): and the best post-GAHT performances were identified for the participants at 21±8 months (range 9-34 months). Post-GAHT times over distances from 60 m to the marathon increased from pre-GAHT times [*p*=0.034, *d*=0.63, 95% CI of difference (87,395)], with an overall increase of 12.7±5.6%. There were time increases of 6.2±1.5% in sprint events, 14.6±3.2% in middle-distance events and 15.7±5.2% in long-distance events.

**Supplemental Table 3.** Best performance times for 6 TW runners within 36 months pre- and post-GAHT (GAHT-3).

| _____________________________________________________________________________ | | | | | | | | |
| --- | --- | --- | --- | --- | --- | --- | --- | --- |
| ID | Distance | Pre | Pre | Pre | Post | Post | Post | Time |
| no. |  | time | Dur | age | time | Dur | age | increase |
|  |  | (h:m:s) | (mos) | (yrs) | (h:m:s) | (mos) | (yrs) | (%) |
| ___________________________________________________________________________ | | | | | | | | |
|  |  |  |  |  |  |  |  |  |
| 3 | 60 m | 8.03 | 10 | 45 | 8.56 | 24 | 47 | 6.6 |
| 3 | 100 m | 12.59 | 7 | 45 | 13.18 | 17 | 47 | 4.7 |
| 3 | 200 m | 24.94 | 9 | 45 | 26.96 | 19 | 47 | 8.1 |
| 3 | 400 m | 56.67 | 6 | 45 | 59.77 | 30 | 48 | 5.5 |
| 4 | 1 mile | 04:09.3 | 2 | 20 | 04:45.8 | 20 | 22 | 14.6 |
| 6 | 1 mile | 04:55.0 | 22 | 44 | 05:26.0 | 28 | 47 | 10.5 |
| 4 | 3000 m | 08:42.3 | 4 | 20 | 09:59.8 | 20 | 22 | 14.8 |
| 9 | 2 miles | 10:25.0 | 6 | 34 | 12:20.0 | 12 | 35 | 18.4 |
| 9 | 5 km | 16:33 | 14 | 33 | 19:11 | 9 | 35 | 15.9 |
| 6 | 5 km | 16:46 | 10 | 45 | 18:48 | 25 | 47 | 12.1 |
| 5 | 5 km | 17:12 | 30 | 39 | 20:25 | 11 | 42 | 18.7 |
| 9 | 5.35 miles | 30:51 | 3 | 34 | 38:24 | 14 | 35 | 24.5 |
| 6 | half mar | 01:17:49 | 9 | 45 | 01:26:16 | 26 | 47 | 10.9 |
| 7 | marathon | 02:48:58 | 5 | 24 | 03:08:50 | 13 | 26 | 11.8 |
| _____________________________________________________________________________ | | | | | | | | |

m=metres; km=kilometres; half mar = half marathon (21.1 km); marathon = 42.2 km; 1 mile = 1609 m; Pre (post) race time = pre (post) GAHT race time in sec(s) or min sec (m:s) or hrs min sec (h:m:s); Pre (Post) Dur = number of months pre (post) GAHT initiation; Pre (Post) age = age in years at which pre (post) GAHT races occurred. Pre (Post) Dur. mos = duration in months pre (post) GAHT
